# Surfactant Protein A in Exhaled Endogenous Particles Is Decreased in Chronic Obstructive Pulmonary Disease (COPD) Patients: A Pilot Study

**DOI:** 10.1371/journal.pone.0144463

**Published:** 2015-12-11

**Authors:** Mona Lärstad, Ann-Charlotte Almstrand, Per Larsson, Björn Bake, Sven Larsson, Evert Ljungström, Ekaterina Mirgorodskaya, Anna-Carin Olin

**Affiliations:** 1 Department of Occupational and Environmental Medicine, Sahlgrenska Academy at University of Gothenburg, Gothenburg, Sweden; 2 Department of Respiratory Medicine and Allergology, Sahlgrenska Academy at University of Gothenburg, Gothenburg, Sweden; 3 Department of Chemistry and Molecular Biology, University of Gothenburg, Gothenburg, Sweden; University of Athens, GREECE

## Abstract

**Background:**

Exhaled, endogenous particles are formed from the epithelial lining fluid in small airways, where surfactant protein A (SP-A) plays an important role in pulmonary host defense. Based on the knowledge that chronic obstructive pulmonary disease (COPD) starts in the small airway epithelium, we hypothesized that chronic inflammation modulates peripheral exhaled particle SP-A and albumin levels. The main objective of this explorative study was to compare the SP-A and albumin contents in exhaled particles from patients with COPD and healthy subjects and to determine exhaled particle number concentrations.

**Methods:**

Patients with stable COPD ranging from moderate to very severe (n = 13), and healthy non-smoking subjects (n = 12) were studied. Subjects performed repeated breath maneuvers allowing for airway closure and re-opening, and exhaled particles were optically counted and collected on a membrane using the novel PExA^®^ instrument setup. Immunoassays were used to quantify SP-A and albumin.

**Results:**

COPD patients exhibited significantly lower SP-A mass content of the exhaled particles (2.7 vs. 3.9 weight percent, p = 0.036) and lower particle number concentration (p<0.0001) than healthy subjects. Albumin mass contents were similar for both groups.

**Conclusions:**

Decreased levels of SP-A may lead to impaired host defense functions of surfactant in the airways, contributing to increased susceptibility to COPD exacerbations. SP-A in exhaled particles from small airways may represent a promising non-invasive biomarker of disease in COPD patients.

## Introduction

Millions of people worldwide suffer from chronic obstructive pulmonary disease (COPD) which is characterized by progressive airflow obstruction that is only partly reversible, inflammation in the airways and systemic effects or comorbidities [1]. Risk factors for developing COPD include long-term exposure to toxic gases and particles, e.g. cigarette smoking and occupational exposures. The disease becomes increasingly prevalent with increasing age [2, 3]. The major sites of disease activity in COPD are small airways (<2 mm in diameter) and lung parenchyma [4–6]. COPD involves a narrowing and eventually an obliteration of the lumen of small airways due to remodeling of the airway wall (bronchiolitis), and destruction of alveolar sacs leading to alveolar airspace enlargement (emphysema) and a loss of functional surface area for gas exchange [7]. Small airways disease in mild COPD probably precedes the development of emphysema [5, 6, 8]. The assessment and monitoring of small airway involvement in COPD is challenging because the region is relatively inaccessible for functional measurements [9, 10]. Improved tools for diagnosing and monitoring disease and a deeper understanding of underlying disease mechanisms are necessary.

Surfactant, a lipoprotein complex, was originally described as being essential for reducing surface tension at the air-liquid interface of the lung, thus preventing collapse of the alveoli. It is now also recognized as a critical component of host immune defense in the lung [11]. Surfactant protein A (SP-A) is the major surfactant protein in the airways and is produced locally, mainly by alveolar type II cells [12]. SP-A is important for enhancing microbial phagocytosis by innate immune cells, such as macrophages and neutrophils, by opsonizing and aggregating bacteria and virus [11]. Epithelial lining fluid (ELF), which contains SP-A, forms an interface between the airspace epithelium and the external environment, and protects against epithelial injury. SP-A is considered a potential biomarker for airway inflammation in respiratory diseases, e.g. in COPD, but studies using different types of human airway samples, such as lung tissue specimens, bronchoalveolar lavage (BAL) and sputum, yield conflicting results [13–18]. Albumin, the most prominent blood protein, is abundant in ELF because of the leakage of plasma proteins from the vasculature into the airways [19]. An increase in albumin can indicate impaired epithelial barrier function.

Only a few non-invasive techniques are presently available for sampling nongaseous particulate matter from central or distal airways: sputum induction and exhaled breath condensate (EBC) sampling [20–26], and sampling of exhaled particles or droplets [27–30]. Sputum is primarily derived from central airways and may sometimes be difficult to obtain. EBC methods could be useful but may present technical and standardization problems, including dilution with water vapor which varies within and between subjects [20–22, 31]. Additionally, methods for measuring exhaled particles and droplets are published, but these do not include chemical quantification [32–34].

A unique and non-invasive method, the PExA^®^ technique, has recently been developed in our department and allows for sampling and measurement of exhaled endogenous particles [35]. The particles originate from the ELF in small airways and are considered to be produced by the airway re-opening following airway closure [36]. Using this method, SP-A levels have previously been quantified in exhaled particles collected from healthy subjects in another study [37], and the data showed good repeatability, with intra- and inter-individual coefficients of variation of 13% and 25%, respectively.

In the present explorative study we aimed to assess, for the first time, the feasibility of the PExA^®^ technique for use with COPD patients. The main goal was to quantify SP-A and albumin content in exhaled particles from COPD patients, as well as particle number concentrations, in order to evaluate whether the levels differed between patients with COPD and healthy subjects.

## Methods

### Subjects and study design

From June 2012 to February 2014 we enrolled physician-diagnosed COPD patients (n = 13, 8 females) with moderate to very severe airflow limitation (GOLD stage 2, n = 2; stage 3, n = 5; stage 4, n = 6) and stable disease. Patients were recruited from an outpatient lung clinic at Sahlgrenska University Hospital, Gothenburg, where they attended regularly. All patients had respiratory medication: inhaled corticosteroids, n = 10; beta_2_-adrenoreceptor agonists, n = 11 (long-acting n = 10); anticholinergics, n = 9 (long acting n = 7); phosphodiesterase-4 inhibitor, n = 1; leukotriene receptor antagonist, n = 1. All medications were permitted during the study. All patients were nonsmokers (12 former smokers; one never-smoker). Patients with a history of lung cancer were excluded. Healthy subjects (n = 12, 8 females) included never-smokers of the same age who regarded themselves as healthy and with no reported respiratory symptoms, no history of pulmonary disease, no medication and with normal spirometry. None of the subjects had signs of respiratory tract infection ≤3 weeks prior to the study. All subjects abstained from eating 1 hour before collection. The study protocol was approved by the Human Research Ethics Committee of the Medical Faculty at University of Gothenburg, Sweden, and all participants gave their oral and written informed consent.

### Lung function tests

Spirometry was performed using a SensorMedics Vmax 22 spirometer (SensorMedics Corp., Yorba Linda, CA, USA). Lung function measurements included forced expiratory volume in one second (FEV_1_), forced vital capacity (FVC) and the FEV_1_/FVC ratio was calculated. Subjects performed at least three technically acceptable trials in accordance with European Respiratory Society (ERS) guidelines [38]. Predicted values were calculated according to the Global Lungs Initiative equations, which also include older ages [39].

### Collection of exhaled particles

The PExA^®^ instrument setup (see S1 Fig) for noninvasive collection of endogenous exhaled particles has been developed in-house as described previously [36], but minor modifications have been made (see supporting information). The subject performs breathing maneuvers via a mouthpiece and a two-way, nonrebreathing valve into a thermostated box (36°C) containing a Grimm 1.108 optical particle counter (Grimm Aerosol Technik GmbH & Co, Ainring, Germany), and an impactor (modified 3-stage PM 10 Impactor, Dekati Ltd., Tampere, Finland) with a Teflon membrane impaction substrate (PTFE, diameter 25 mm; Merck Millipore Ltd., Cork, Ireland). The impactor was modified to reduce the particle cut-off diameter and thus increase the amount of collected material from airways. Using a vacuum pump the exhaled air containing particles is drawn through the impactor and the particles are collected by impaction according to size on the membrane. The particle number concentration and size distribution are measured by light scattering using the optical particle counter, operating on the sample stream with 1 s time resolution. The instrument was calibrated using monodisperse, spherical particles of known physical size and refractive index. The particle sizes were recalculated taking into account the different refractive index [40]. The measured particle sizes covered diameters between 0.41 and 4.55 μm sorted into the following bin sizes: 0.41–0.55, 0.55–0.70, 0.70–0.92, 0.92–1.14, 1.14–1.44, 1.44–2.36, 2.36–2.98, 2.98–4.55 μm.

Subjects inhaled HEPA-filtered (Whatman Inc., NJ, USA) air for 2 min before the sampling in order to remove particles originating from ambient air. All subjects wore a nose clip throughout the procedure. The subjects were instructed to perform the following standardized breathing maneuvers to allow for airway closure and re-opening: i) exhale fully to residual volume and hold breath for three seconds, ii) inhale rapidly to vital capacity, iii) exhale normally, iiii) breathe tidally until particle concentration is <150 particles/L. Only the exhalation in iii was sampled in the instrument. An ultrasonic flow sensor (OEM flow sensor, Spiroson-AS, Medical Technologies, Zürich, Switzerland) measured flow-rates. The inhalation and exhalation flow-rate was displayed graphically in real-time on a computer screen, which helped the subjects to perform the required breathing maneuvers. Between breathing maneuvers, the test subject breathed particle-free air tidally. The procedure was repeated until a target sample volume of 60 L was reached. After collection the Teflon membrane was immediately transferred to a low-binding Eppendorf polypropylene vial and stored at -80°C until analysis. During the study a negative control sample was included which consisted of a Teflon membrane placed in the PExA^®^ instrument for 30 min during a run but without collection of exhaled particles. The negative control sample was treated in the same way as the other samples.

### Sample desorption and immunodetection

Desorption of particles from Teflon membranes was performed by adding 140 μl of phosphate-buffered saline (PBS; 10 mM sodium phosphate, 15 mM sodium chloride) containing 1% bovine serum albumin (BSA) and 0.05% Tween-20 to each sample vial and shaking at 400 rpm during 60 minutes at 37°C in a thermomixer (Thermomixer comfort; Eppendorf AG, Hamburg, Germany). Particle contents of SP-A and albumin were quantified according to the manufacturer´s instructions using a human SP-A enzyme-linked immunosorbent assay (ELISA) kit from BioVendor Laboratorní medicína a.s. (Brno, Czech Republic) and a human albumin ELISA kit from Immunology Consultants Laboratory, Inc. (Portland, OR, USA), respectively. Prior to ELISA, all samples were diluted 3 times with ELISA manufacturer’s diluent, resulting in a final manufacturer’s diluent/desorption solution ratio of 2:1 (v:v). Samples with mass >500 ng were further diluted 3 times. All calibrants were directly prepared using manufacturer´s diluent/desorption solution. Absorbance was measured at 450 nm by a plate-reader (BioTek ELx-808UI; Highland Park, MI, USA). Limits of detection (LOD) of the SP-A and albumin assays were calculated by using instrument blanks (n = 6) that were prepared identically to the participants samples, but without exhalation into the instrument. LOD (mean_blank_ + 3*standard deviation_blank_) were found to be 0.09 ng/ml for SP-A and 0.79 ng/ml for albumin (corresponding to 0.04 and 0.33 ng/sample, respectively). All SP-A and albumin levels in exhaled particle samples were above LOD. The negative control sample had undetectable amounts of SP-A and albumin, i.e. below LOD. Intra-assay variability was 3.1% and 2.6% for SP-A and albumin assays, respectively.

### Calculation of mass and concentrations

Total mass of collected particles was calculated based on the number concentration and the size of the particles, as measured by the Grimm instrument. The particles were assumed to be spherical and have a density of 1000 kg/m^3^. Particle mass concentrations were defined as total particle mass divided by total exhaled volume (ng/L). SP-A and albumin concentrations were quantified in desorbed samples and expressed both as mass concentrations, i.e. mass divided by total exhaled volume (pg/L), or as weight percent (wt%), i.e. the mass divided by the total particle mass and expressed as a percentage.

### Statistical analysis

Data are summarized as mean (SD) for normally distributed variables and as median (interquartile range) for non-normal distributed variables. For normally distributed data, the two-sided Student’s t-test was used, and for non-normal data, the two-sided, nonparametric Mann Whitney U test was used. Spearman rank correlations (r_s_) were used to test for linear relationships between variables. A p-value of <0.05 was used to define statistical significance. Data were analyzed using version 9.3 of the SAS statistical software package (SAS Institute Inc., Cary, NC, USA).

## Results

### Demographic and clinical data

Basic characteristics of the study subjects are presented in Table 1. COPD patients had significantly lower lung function compared to healthy subjects. There were no significant differences regarding age or BMI.

**Table 1:** Demographic and clinical data of healthy subjects and COPD patients.

	Healthy subjects	COPD patients	p-value
**Females/males n**	8/4	8/5	
**Age years**	68.3 ± 9.3	71.4 ± 6.5	0.35
**BMI kg/m** ^**2**^	24.6 ± 2.3	27.9 ± 5.5	0.066
**FVC L**	3.4 ± 0.7	2.6 ± 0.8	0.011
**FVC % pred**	101.5 ± 10.9	77.4 ± 14.8	0.0001
**FEV**_**1**_ **L**	2.7 ± 0.5	1.0 ± 0.6	<0.0001
**FEV**_**1**_ **% pred**	103.1 ± 10.0	38.5 ± 16.0	<0.0001
**FEV**_**1**_ **/FVC %**	78.3 ± 5.5	37.5 ± 12.2	<0.0001
**FEV**_**1**_ **/FVC % pred**	101.0 ± 8.5	48.9 ± 16.6	<0.0001
**Smoking history pack-years** ^a^	0	37.2 ± 21.0	<0.0001

Data are presented as mean ± SD unless otherwise stated; COPD: chronic obstructive pulmonary disease; BMI: body mass index; L: liter; FVC: forced vital capacity; FEV_1_: forced expiratory volume in 1 s. % pred: % predicted, according to the Global Lungs Initiative equations [39].

^a^ COPD patients: 12 former smokers and one never-smoker.

### Exhaled particles

**Fig 1:**
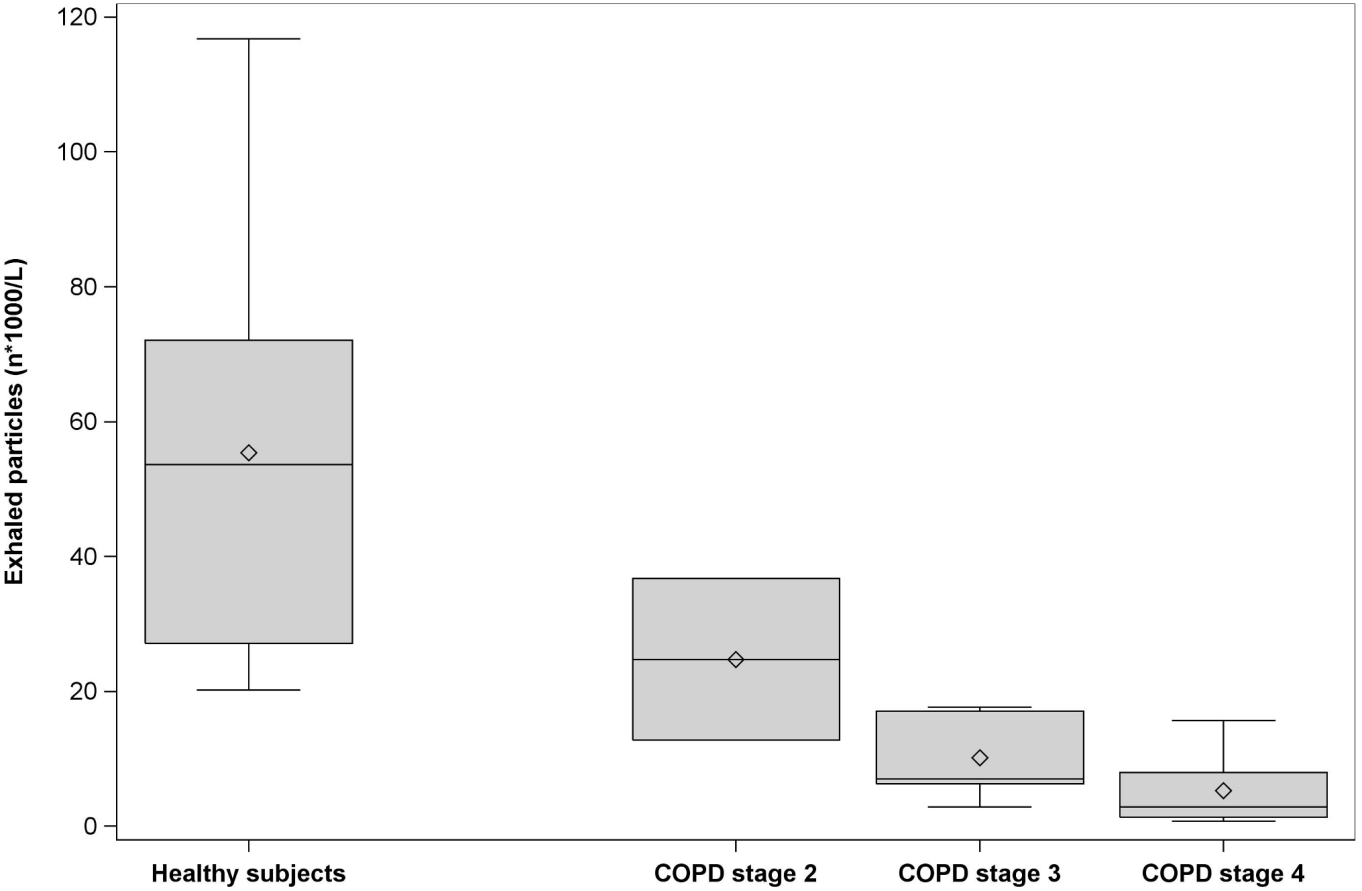
Box plots of particle number concentrations among healthy subjects and among COPD patients according to stage. Healthy subjects (n = 11) and patients with COPD stage 2 (n = 2), stage 3 (n = 5) and stage 4 (n = 6) were included. Horizontal lines represent the median, diamond shapes represent the mean, boxes represent the interquartile range and whiskers represent the range.

COPD patients had substantially lower particle number concentration, number of particles per exhalation and mass concentration of all particle sizes compared to healthy controls (Table 2) and particle number concentration decreased with increasing COPD stage (Fig 1).

**Table 2:** Exhaled particle data.

Variable	Healthy subjects (n = 12)			COPD patients (n = 13)			p-value
	Median	Lower Quartile	Upper Quartile	Median	Lower Quartile	Upper Quartile	
**Particle number concentration** ^a^ n*1000/L	53.6	27.1	72.1	7.0	2.8	15.7	<0.0001
**Particle number concentration** ^a^ n*1000/exhalation	160.1	74.8	194.2	14.1	5.3	39.4	0.0002
**Particle mass concentration ng/L**	9.2	5.8	13.9	1.1	0.4	2.8	0.0004

Data are presented as median, with interquartile range. The particle number and mass concentrations represent particle diameters from 0.41 to 4.55 μm. Particle number concentrations should be multiplied by 1000 (i.e. n*1000). L: liter; ng/L: ng per liter exhaled air.

^a^Number concentration data are missing for one healthy subject.

**Fig 2:**
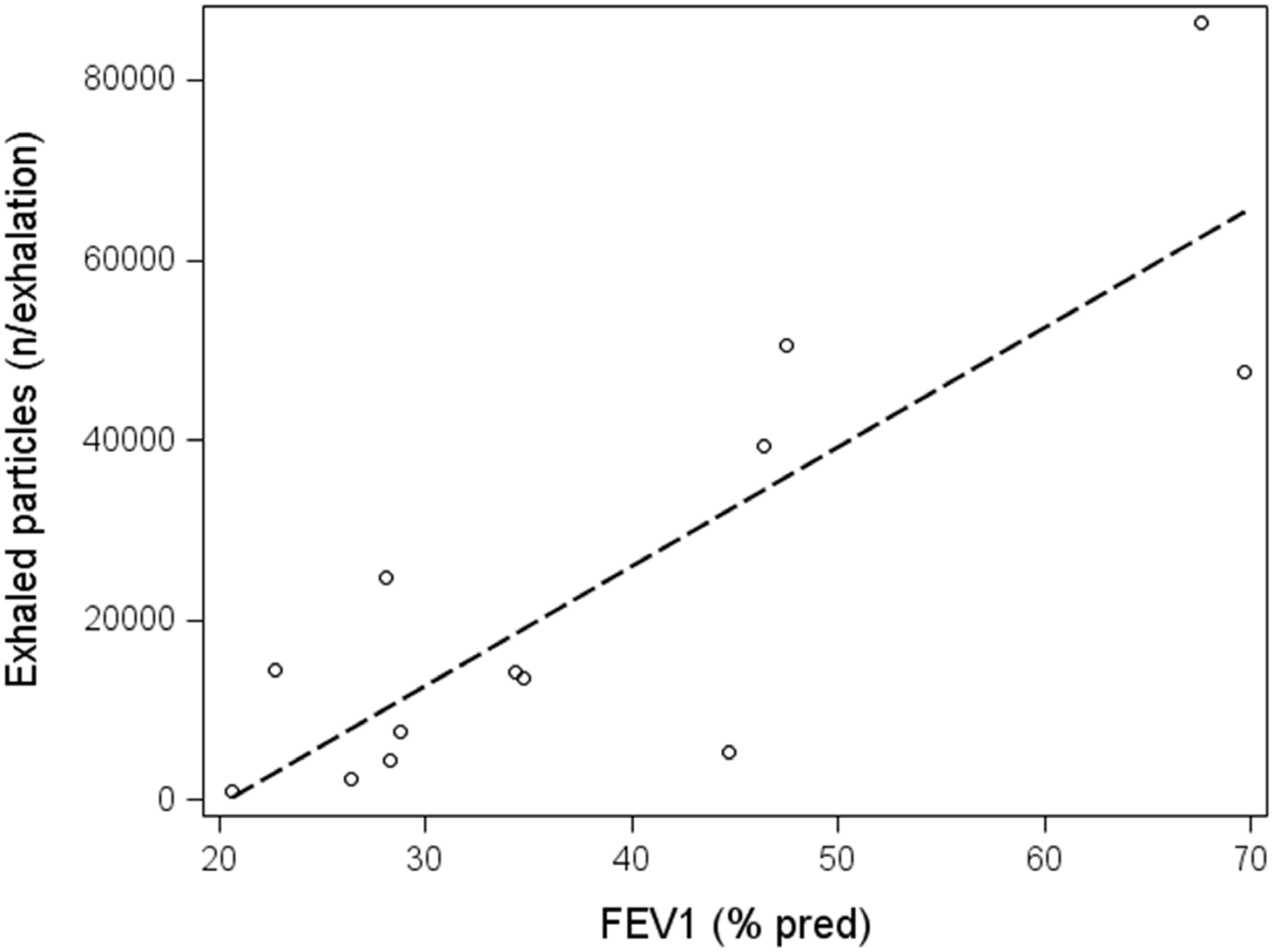
Correlation between FEV_1_% pred and exhaled particles per exhalation. There was a significant association between FEV_1_% pred and exhaled particles per exhalation among COPD patients which was fairly high (r_s_ = 0.72; p = 0.0055). The corresponding association among healthy subjects was not significant (r_s_ = 0.073; p = 0.83).

The strongest correlation was found between number of particles per exhalation and FEV_1_% pred among COPD patients (Fig 2), but there were also significant correlations between number of particles per exhalation and FEV_1_/FVC % (r_s_ = 0.71, p = 0.0071) and FEV_1_/FVC % pred (r_s_ = 0.69, p = 0.0095). Additionally, there were significant correlations between particle number concentration and FEV_1_% pred (r_s_ = 0.66, p = 0.014), FEV_1_/FVC % (r_s_ = 0.69, p = 0.0092) and FEV_1_/FVC % pred (r_s_ = 0.66, p = 0.013). There were no significant correlations between particle number concentration and spirometry variables among healthy subjects apart from particle mass concentration which correlated negatively with FEV_1_/FVC % pred (r_s_ = -0.66, p = 0.019).

### SP-A and albumin in exhaled particles

SP-A and albumin mass concentrations were substantially lower in COPD patients compared to healthy subjects (Table 3). SP-A mass concentrations correlated strongly with particle number and particle mass concentrations in the whole group, i.e. in both COPD patients and healthy subjects (r_s_ = 0.97 and 0.97, respectively; p<0.0001), and this was also the case regarding albumin (r_s_ = 0.94 and 0.95, respectively; p<0.0001). Moreover, SP-A weight percent (wt%, mass corrected for total particle mass) was significantly lower in COPD patients compared to healthy subjects, whereas albumin wt% was not significantly different. The ratio of SP-A to albumin was not different between groups.

**Table 3:** SP-A and albumin concentrations in exhaled particles.

Variable	Healthy subjects			COPD patients			p-value
	Median	Lower Quartile	Upper Quartile	Median	Lower Quartile	Upper Quartile	
**SP-A mass concentration pg/L**	321	250	458	35	12	115	0.0004
**SP-A wt%**	3.9	3.0	4.8	2.7	2.5	3.5	0.036
**Albumin mass concentration pg/L**	494	318	956	60	22	161	0.0007
**Albumin wt%**	6.5	4.7	8.5	6.3	5.4	7.0	0.57
**SP-A/albumin**	0.6	0.5	0.7	0.5	0.3	0.7	0.37

Data are presented as median, with interquartile range. L: liter, pg/L: pg per liter exhaled air, wt%: weight percent.

**Fig 3:**
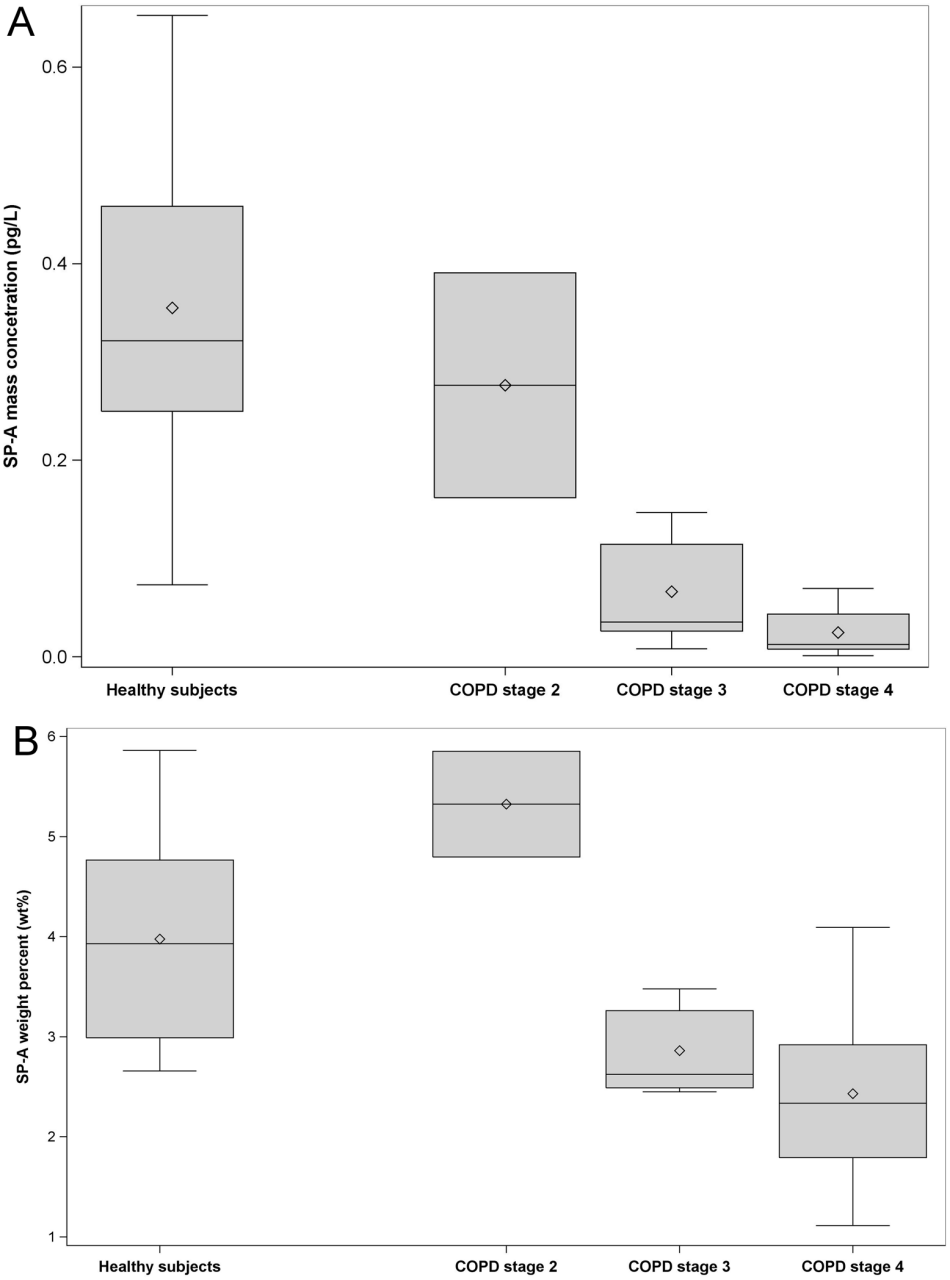
Box plots of SP-A concentrations among healthy subjects and COPD patients according to stage. Panel (A) shows SP-A mass concentration (pg/L) and panel (B) shows SP-A weight percent (wt%). Healthy subjects (n = 11) and patients with COPD stage 2 (n = 2), stage 3 (n = 5) and stage 4 (n = 6) were included. Horizontal lines represent the median, diamond shapes represent the mean, boxes represent the interquartile range and whiskers represent the range.

SP-A mass concentrations decreased with increasing COPD stage (Fig 3A). SP-A wt% values were significantly decreased (p = 0.0042) in COPD patients with stage 3 and 4 (n = 11)(Fig 3B) compared to healthy subjects. There was a trend to increased SP-A wt% values in COPD stage 2, but only two patients were included in this group.

**Fig 4:**
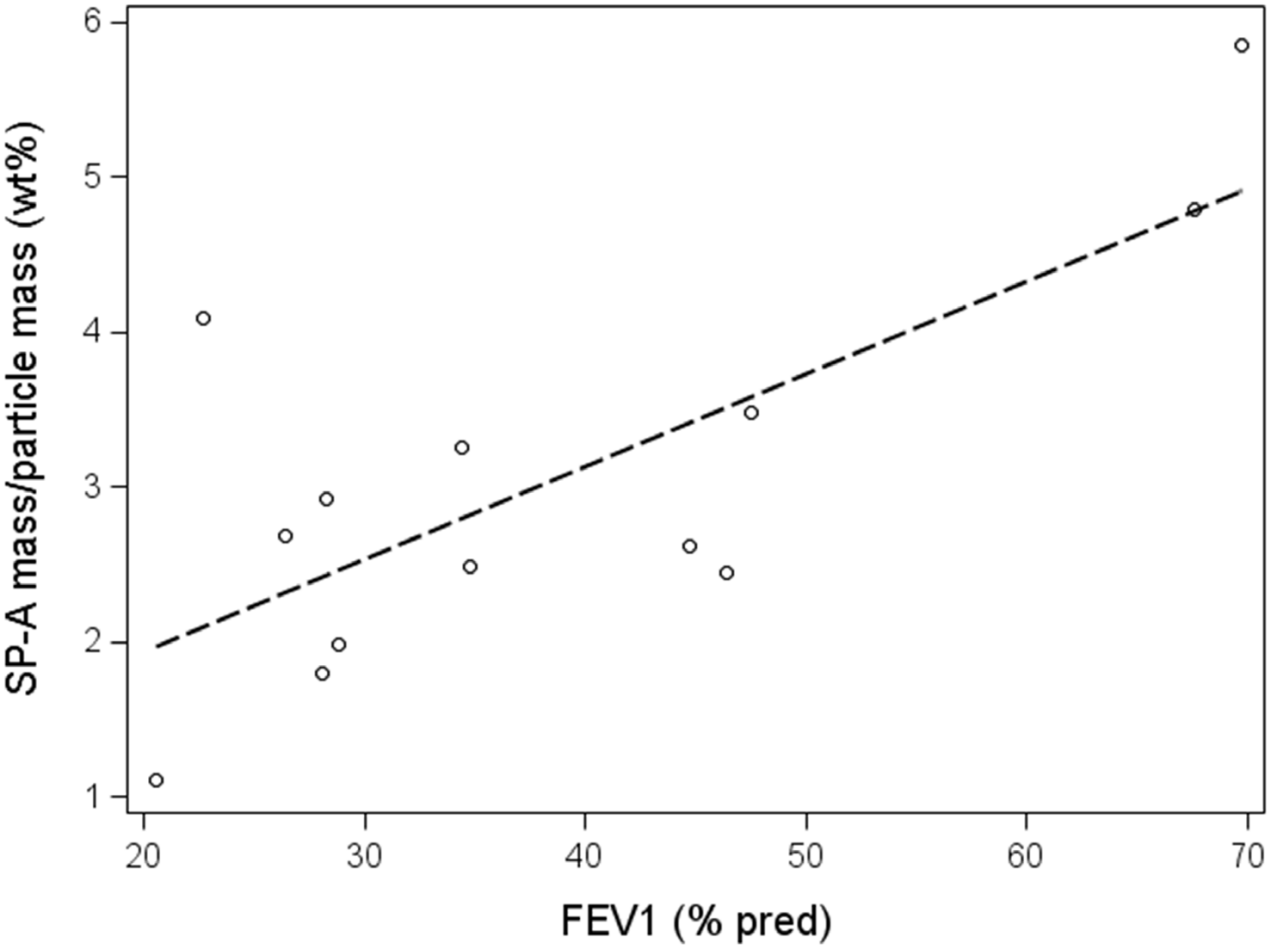
Correlation between FVC % pred and SP-A weight percent. There was a significant association between FVC % pred and SP-A weight percent wt% (r_s_ = 0.*57*; p = 0.041) among COPD patients. The corresponding association among healthy subjects was not significant (r_s_ = -0.42; p = 0.17).

The correlation between SP-A wt% and lung function variables was significant for FVC % pred among COPD patients (Fig 4). The correlation between SP-A wt% and the other spirometry variables did not reach significance. There were no significant associations between SP-A wt% and spirometry variables among healthy subjects.

When including both healthy subjects and COPD patients, SP-A mass concentrations correlated significantly to all of the spirometry variables (r_s_ range: 0.69 to 073; all p-values <0.0001 except for FVC % pred, p = 0.0001). In all subjects SP-A wt% correlated significantly to all of the spirometry variables (r_s_ range: 0.54 to 0.56; p-values, range: 0.0034 to 0.0056) except for FVC % pred (r_s_ = 0.39, p = 0.057).

### Breathing maneuver

COPD patients needed slightly higher number of exhalations compared to healthy subjects to reach an exhaled air volume of 60 L (mean n = 29 ± SD 7.2, vs. n = 25 ± SD 8.9, respectively). The collection time was approx. 20 to 30 minutes, including short pauses when needed. COPD patients needed more pauses during the procedure compared to healthy subjects (median 2 vs. 0.5, respectively) and they needed approx. 30% more time. Six of the healthy subjects did not take pauses, compared to two of the COPD patients. One COPD patient and one healthy subject felt that the repeated breathing maneuvers were very strenuous and reached only 45 L and 42.5 L, respectively. The results were similar when these two subjects were excluded. One COPD patient (73 year old female, GOLD stage 2) did not manage to complete the sampling and was excluded from the study. In conclusion, almost all COPD patients were able to successfully perform the maneuvers.

## Discussion

We have demonstrated that collection of exhaled particles originating from small airways using the novel and unique PExA^®^ technique followed by particle analysis provides a non-invasive and feasible way to examine and monitor ELF composition in COPD patients. We found that SP-A content (weight percent) in exhaled particles was significantly lower in COPD patients than in healthy subjects. SP-A content decreased in exhaled particles with increased disease severity, while albumin content was similar for all levels of disease severity. Furthermore, COPD patients exhaled substantially fewer particles than healthy subjects.

Mass concentrations of SP-A and albumin are directly related to the amount of sampled ELF, which explains the strong correlation between each of the mass concentrations and the exhaled particle mass. Thus, a low SP-A mass concentration does not necessarily imply a small amount of SP-A in the ELF, but may instead reflect a low total amount of particles. However, a reduced SP-A weight percent of SP-A in a sample indicates reduced SP-A levels in the ELF and thus presents a noteworthy finding.

Particle number and mass concentrations were considerably lower in COPD patients than in healthy subjects. In principal this may be due to decreased production of particles and/or increased loss of produced particles. Particle number correlated strongly and positively to FEV_1_% pred. This measure is usually considered to reflect large airways but may of course also be related to the degree of obstruction of small airways. Presumably the reduced lung function in the COPD patients reflects a reduced number of airways available for closure and opening and an increased residual volume. Bronchiolitis and emphysema may result in decreased number of small airways that can be closed and reopened. Moreover, the inflammatory processes in the small airways and alveoli may alter the relevant mechanical properties, causing airway closure and opening to occur at a more proximal localization. Translocation to one more proximal airway generation reduces the number of available airways by 50% because of the airway branching dichotomy. Furthermore, the viscoelastic properties of the ELF may be altered such that fewer airways close and re-open and/or fewer particles are produced during airway opening. Reduced elastic properties of elements within the airway wall may affect airway recoil, leading to decreased particle formation. Additionally, increased loss of particles in the airways during exhalation due to increased particle impaction may occur because of dynamic compression of airways, since airflow limitation and dynamic compression may take place even during spontaneous breathing in COPD patients [41]. Increased loss of particles by impaction may also be due to decreased exhalation flowrate. In conclusion, several mechanisms may operate together to reduce the amount of particles exhaled by COPD patients. Importantly, the relative particle concentration (wt% for patient or healthy control) should be independent of the number of exhaled particles and the breathing maneuver, and could thus act as a biomarker.

Hypothetically, lower SP-A weight percent levels in the COPD patients may be due to increased consumption and degradation of SP-A due to chronic airway inflammation and oxidative stress, reduced cellular expression and/or increased leakage of SP-A into the circulation. SP-A levels have previously been quantified in various airway samples, but the results diverge. Some studies show significantly reduced SP-A levels in BAL fluids from smokers with or without emphysema compared to non-smokers [13, 14]. SP-A levels in lung tissue specimens from COPD patients and smokers without COPD were significantly lower than in specimens from non-smoking controls [18]. On the contrary, another study of human lung tissue revealed increased levels of SP-A in COPD patients, but not in the normal lung [17]. Furthermore, elevated SP-A protein levels were detected in the induced sputum supernatants from COPD patients. Ishikawa et al. showed that patients with COPD had higher levels of sputum SP-A than subjects with prolonged cough [15]. Mazur et al. found that sputum SP-A was non-detectable in most non-smokers, but drastically increased in subjects with smoking habits and airway ailments [16]. Altogether, these studies demonstrate that available data regarding SP-A levels in in lung tissue from COPD patients are conflicting, and that SP-A levels appear elevated in sputum but reduced in BAL. BAL is believed to derive from peripheral airways, and the findings in BAL corroborate our own findings in exhaled particles.

SP-A levels (weight percent) in exhaled particles from stage 2 COPD patients were increased compared to healthy subjects (Fig 3B). There were, however, only two subjects in this group and therefore no conclusions can be drawn from this result. In light of the importance of SP-A in the host immune defense against pathogens, reduced SP-A levels in more severe COPD might contribute to increased frequency of airway infections in COPD patients [42].

Albumin levels (weight percent) were not significantly altered in exhaled particles from COPD patients, indicating that albumin amounts in patient ELF may not differ from those in healthy subjects. Henriksen et al. found no difference in *overall* albumin leakiness between COPD patients and controls after intravenous injections of radio-iodinated albumin [43]. Minakata et al., however, found a correlation between normalized airway albumin (sampled by a probe from ELF) and FEV_1_ percent predicted in COPD patients [44]. Other findings in BAL and sputum specimens similarly diverge [45–48], although plasma exudation is suggested as a feature of COPD [49].

Patients with COPD had a lung physician’s diagnosis that was confirmed by spirometry for a median period of 9 years prior to the study. All but one patient had a history of tobacco smoking; however passive smoking was not investigated. It is known that most patients with COPD have different degrees of emphysema, in combination with chronic bronchitis. Yet, the degree of emphysema was not evaluated in this study. We did not compare our results to an established technique such as BAL which also reflects the small airways. BAL is an invasive method that may have problems with sample dilution resulting in low reproducibility and difficulties in interpretation.

COPD is a heterogeneous disease; ideally our study would include more subjects at different COPD stages, as well as smokers without COPD. COPD patients were recruited via an outpatient lung clinic at a hospital, and therefore only patients with moderate to very severe COPD were included. Despite the limitations with this explorative study, we were able to show significantly reduced levels of SP-A and particle number concentrations in patients with COPD compared to healthy subjects.

In conclusion, quantifying and characterizing exhaled particles comprise a promising method for examining small airway ELF, where host immune defense in COPD patients appears attenuated. Specifically, SP-A may be used as a biomarker to identify and monitor COPD. Decreased SP-A levels may imply impaired host defense functions of surfactant in the airways.

## Supporting Information

S1 Fig Schematic outline of the instrument setup. The subject performs breathing maneuvers via a mouthpiece and a directional, nonrebreathing valve (A) into a thermostated box (36°C)(B) containing a modified 3-stage impactor (C) with a Teflon (PTFE) membrane. Particle-free air is inhaled through a HEPA filter (D) to avoid interference with background aerosol. Exhaled particles are sampled on the membrane by impaction according to size. The particle concentration is measured by an optical particle counter (E) by using a side sample stream of 20 ml/s from the reservoir (F) that serves as a buffer when the flow of exhaled air from the subject exceeds the combined impactor and particle counter flows. The vacuum pump (G) draws a flow of 250 ml/s from the reservoir through the impactor. A respiratory humidifier (H) is used to humidify compressed air (I). A flow-sensor (J) measures the flow-rate. Inhalation and exhalation flow-rates are displayed graphically in real-time on a computer screen (K). Minor modifications to the setup have been made compared to the setup that was used by Almstrand et al. (2010): a Teflon membrane was included and a respiratory humidifier (MR730, Fisher & Paykel Healthcare Ltd) was used.
(TIF)
