# Supplementary figures and images for: Surfactant Protein A in Exhaled Endogenous Particles Is Decreased in Chronic Obstructive Pulmonary Disease (COPD) Patients: A Pilot Study
Source: PLoS One. 2015 Dec 11;10(12):e0144463. doi: 10.1371/journal.pone.0144463 (PMC4676630; doi:10.1371/journal.pone.0144463)

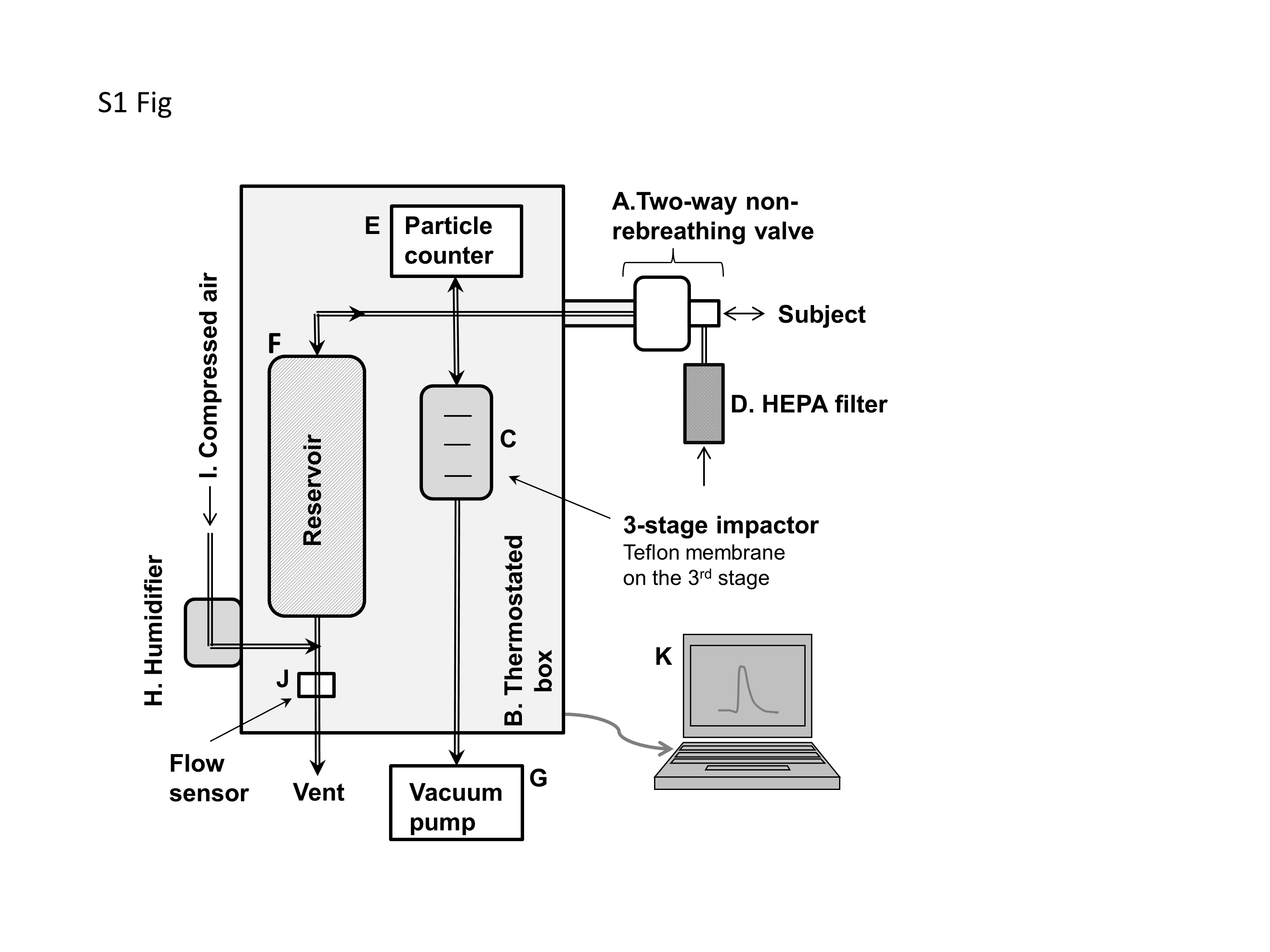

Supplement: S1 Fig — The subject performs breathing maneuvers via a mouthpiece and a directional, nonrebreathing valve (A) into a thermostated box (36°C)(B) containing a modified 3-stage impactor (C) with a Teflon (PTFE) membrane. Particle-free air is inhaled through a HEPA filter (D) to avoid interference with background aerosol. Exhaled particles are sampled on the membrane by impaction according to size. The particle concentration is measured by an optical particle counter (E) by using a side sample stream of 20 ml/s from the reservoir (F) that serves as a buffer when the flow of exhaled air from the subject exceeds the combined impactor and particle counter flows. The vacuum pump (G) draws a flow of 250 ml/s from the reservoir through the impactor. A respiratory humidifier (H) is used to humidify compressed air (I). A flow-sensor (J) measures the flow-rate. Inhalation and exhalation flow-rates are displayed graphically in real-time on a computer screen (K). Minor modifications to the setup have been made compared to the setup that was used by Almstrand et al. (2010): a Teflon membrane was included and a respiratory humidifier (MR730, Fisher & Paykel Healthcare Ltd) was used. (TIF) [file pone.0144463.s001.tif]
